# The burden of HIV on Tuberculosis patients in the Volta region of Ghana from 2012 to 2015: implication for Tuberculosis control

**DOI:** 10.1186/s12879-017-2598-z

**Published:** 2017-07-19

**Authors:** Eric Osei, Joyce Der, Richard Owusu, Philip Kofie, Wisdom Kudzo Axame

**Affiliations:** 1 Department of Population and Behavioural Sciences, School of Public Health, University of Health and Allied Sciences, Hohoe, Ghana; 2 Department of Epidemiology and Biostatistics, School of Public Health, University of Health and Allied Sciences, Hohoe, Ghana; 3 Department of Family and Community Health, School of Public Health, University of Health and Allied Sciences, Hohoe, Ghana

**Keywords:** TB, HIV, Co-infection, Burden, Volta region, Ghana

## Abstract

**Background:**

The impact of HIV on TB, and the implications for TB control, has been acknowledged as a public health challenge. It is imperative therefore to assess the burden of HIV on TB patients as an indicator for monitoring the control efforts of the two diseases in this part of the world. This study aimed at determining the burden of HIV infection in TB patients.

**Methods:**

We conducted a retrospective review of TB registers in five districts of the Volta Region of Ghana. Prevalence of TB/HIV co-infection was determined. Bivariate and multivariate logistic regression were used to identify the predictors of HIV infection among TB patients and statistical significance was set at *p*-value <0.05.

**Results:**

Of the 1772 TB patients, 1633 (92.2%) were tested for HIV. The overall prevalence of TB/HIV co-infection was (18.2%; 95% CI: 16.4–20.1). The prevalence was significantly higher among females (24.1%; 95%CI: 20.8–27.7), compared to males (15.1%; 95% CI: 13.1–17.4) (*p* < 0.001) and among children <15 years of age (27.0%; 95% CI: 18.2–38.1), compared to the elderly ≥70 years (3.5%; 95% CI: 1.6–7.4) (*p* < 0.001). Treatment success rate was higher among patients with only TB (90%; 95% CI: 88.1–91.5) than among TB/HIV co-infected patients (77.0%; 95% CI: 71.7–81.7) (*p* < 0.001). Independent predictors of HIV infection were found to be: being female (AOR: 1.79; 95% CI: 1.38–2.13; *p* < 0.001); smear negative pulmonary TB (AOR: 1.84; 95% CI: 1.37–2.47; *p* < 0.001); and patients registered in Hohoe, Kadjebi, and Kpando districts with adjusted odds ratios of 1.69 (95% CI: 1.13–2.54; *p* = 0.011), 2.29 (95% CI: 1.46–3.57; *p* < 0.001), and 2.15 (95% CI: 1.44–3.21; *p* < 0.001) respectively. Patients ≥70 years of age and those registered in Keta Municipal were less likely to be HIV positive with odds ratios of 0.09 (95% CI: 0.04–0.26; *p* < 0.001) and 0.62 (95% CI: 0.38–0.99; *p* = 0.047) respectively.

**Conclusion:**

TB/HIV co-infection rate in five study districts of the Volta region is quite high, occurs more frequently in female patients than males; among smear negative pulmonary TB patients, and children <15 years of age. Findings also demonstrate that HIV co-infection affects TB treatment outcomes adversely. Strengthening the TB/HIV collaborative efforts is required in order to reduce the burden of co-infection in patients.

## Background

Tuberculosis (TB) has existed for years and remains a major global public health problem. It causes ill-health in millions of people each year and in the year 2015, TB was among the top 10 causes of death worldwide, ranking above Human Immunodeficiency Virus/Acquired Immune Deficiency Syndrome (HIV/AIDS) as one of the leading causes of death from an infectious disease [1]. The 2015 estimates of the World Health Organization (WHO) showed that there were 10.4 million incident cases of TB worldwide of which, about 10% were co-infected with Human immuno-deficiency Virus (HIV) and about 1.4 million deaths, of which, 400,000 deaths were among people co-infected with HIV [1].

The HIV pandemic represents an important challenge to global TB control [2]. Many sub-Sahara African countries have borne the impact of the generalised HIV and tuberculosis epidemics, which have strained health systems and devastated populations in the region. Overall, 32% of TB cases were estimated to be co-infected with HIV in this region, which represents 74% of TB cases among people living with HIV worldwide [1].

TB/HIV co-infection poses widespread diagnostic, management and economic challenges. HIV infection has been reported to be associated with an increased risk of developing active TB by facilitating disease progression during primary TB infections or reactivation of latent infection by 20-fold [3, 4]. On the other hand, TB is a leading cause of death among people living with HIV [5], and TB/HIV co-infected patients, especially in the absence of antiretroviral therapy (ART), have significantly worse prognosis [6]. Additionally, HIV infection is associated with atypical clinical presentation of TB including smear negative pulmonary TB, normal chest X-ray and higher frequency of extra-pulmonary TB (EPTB); thus, challenging TB diagnosis in middle and low-income countries [7]. It is imperative therefore to periodically estimate the burden of TB/HIV co-infection as an indicator for monitoring the control efforts of the two diseases in this part of the world.

In Ghana, as in other sub-Saharan African countries, TB remains a major public health problem.

In 2015, the national prevalence was estimated at 356 per 100,000 population and incidence rate of 160 per 100,000 of the population [1].

The impact of HIV on TB, and the implications for TB and HIV control, have been acknowledged as a public health challenge in Ghana, as is the case in many other Sub-Saharan African countries. WHO classifies Ghana as having high burden of TB/HIV co-infection. According to the WHO, there were approximately 9900 incident cases of TB among HIV positive patients representing a rate of 36 per 100, 000 of the Ghanaian population in 2015 [1]. There has not been a systematic, nationwide study to analyse the prevalence of TB/HIV co-infection in Ghana. However, estimates show that the influence of HIV on TB has been increasing since 1989 while about 14% of TB cases could be attributed to HIV/AIDS. Hospital studies have also shown that the prevalence of HIV in TB patients is approximately 25–30% and that as many as 50% of patients with chronic cough could be HIV positive (Ghana Health Services: Guildelines for the clinical management of TB and HIV Co-infection in Ghana, unpublished).

Major progress has been made in global TB control following the widespread implementation of the Directly Observed Treatment Short-course (DOTS) strategy in many countries [8]. Full implementation of this strategy is however inadequate to control TB where HIV is fuelling the TB epidemic, and control of HIV infection must therefore become an important concern for National TB Programmes (NTP). In view of this, WHO updated its 2004 policy guideline that recommends offering routine HIV testing to patients with presumptive or diagnosed TB as a means of reducing the burden of HIV [2].

Since, June 2007, TB/HIV activities have been incorporated into the existing TB and HIV services in Ghana by ensuring routine ascertainment of HIV status in people with TB and vise visa in many health facilities and some communities in Ghana. Antiretroviral Therapy (ART) for all people with TB/HIV co-infection is also provided in all public and some private hospitals in the country as part of basic services for people living with TB/HIV [9].

In view of the rapidly changing face of the HIV epidemic and the emergence of an epidemic of TB/HIV co-infection, there is a pressing need for continuous evaluation of the magnitude of HIV infections among TB patients to help guide policies and strategies for scaling up TB/HIV collaboration activities. To this end, the current study aimed at assessing the burden of HIV infection among TB patients in the Volta Region of Ghana and to describe the association between HIV infection and characteristics of TB patients to provide a firm evidence base to underpin the planning and improvement of future collaborative TB/HIV activities.

## Methods

### Study settings and design

**Fig. 1:**
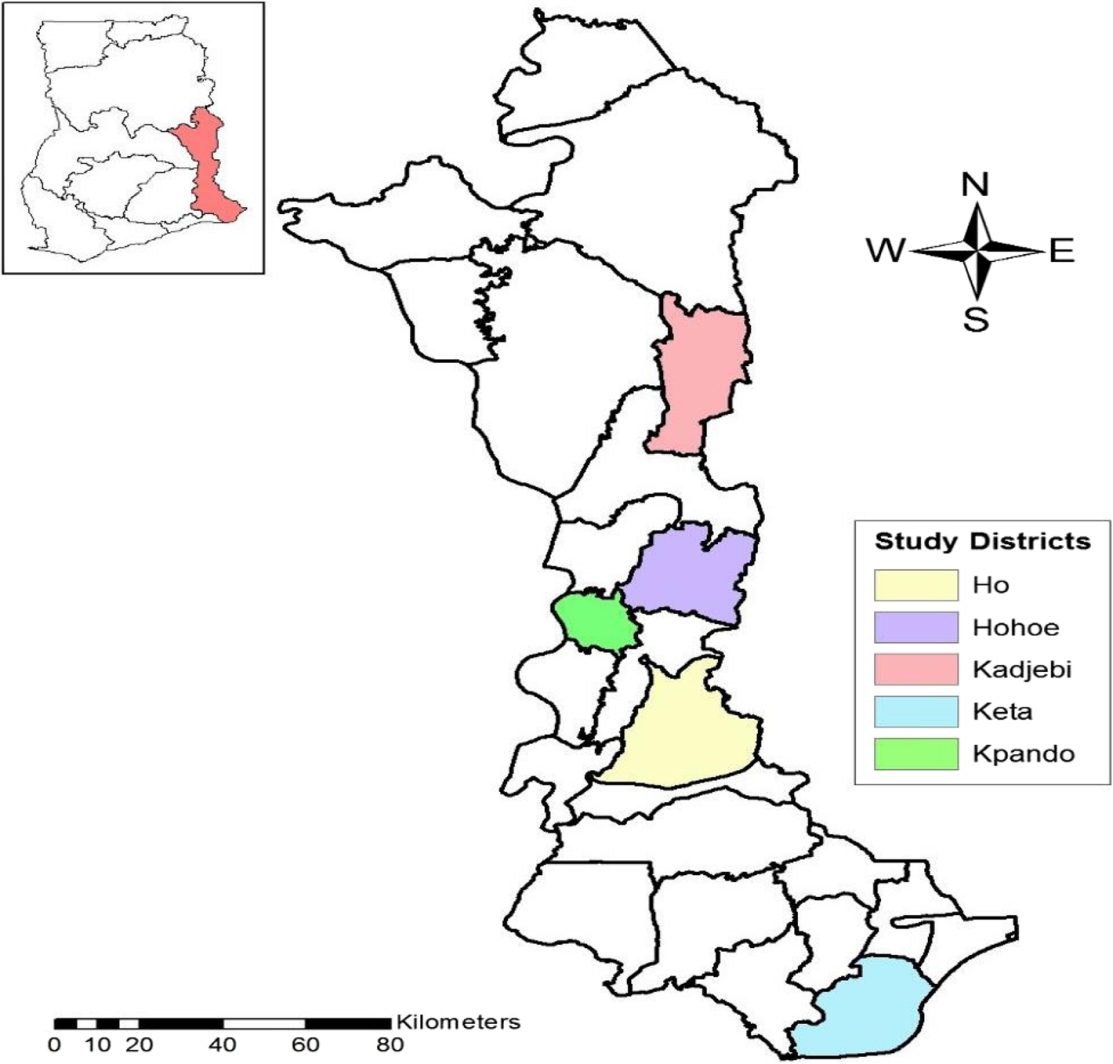
Map of Volta Region showing study areas

We conducted a retrospective study of all TB cases registered from 2012 to 2015 in five districts of Volta Region of Ghana. The districts were Ho, Hohoe and Kpando in the Middle zone; Kadjebi in the Northern zone and Keta in the Southern zone of the Volta region. Three districts were selected from the middle zone due to high population density compared with the other zones. The Volta region is the longest region in Ghana, and stretches from the Gulf of Guinea through nearly all the vegetational zones found in the country. The Region is divided into 25 administrative districts/municipalities with Ho as the Regional capital. Volta Region has a total of 377 health facilities serving a population of 2,789,211 with a growth rate of 2.5%. The region is divided into three natural geographical belts namely the southern, middle and the northern belts. There are 44 DOTS centres and 41 diagnostic centres in the Region that provide TB diagnosis and treatment services to suspected clients. TB diagnosis, treatment, and monitoring are done as per the national tuberculosis control program (NTBCP) guidelines (Volta Regional Health Directorate: 2015 annual report, unpublished). All patients diagnosed with TB are also supposed to undergo HIV counselling and testing. Fig. 1 represents the map of the Volta region showing the study districts.

### Data source

Data were extracted from the district TB register (TB 07). The register is a standardized document used by all district tuberculosis control programmes in Ghana. It is kept by the district TB focal person who goes round all treatment centres within the catchment area from time to time to update it by serially transferring patients’ information from the facility TB registers into the district register. These information include patients’ demographics, identifiers, type and category of TB, initial and follow up sputum smear examination results, HIV test results, and treatment outcomes.

### Data handling and analysis

Extracted data were entered into Microsoft Excel spread sheet for cleaning and exported into Stata Version 11.0 (Stata Corp, College Station, Texas, USA) for analysis. The excel spread sheet was designed to capture all important variables from the registers such as Age, Sex, Patient Type, Disease classification, Sputum Smear result, Treatment outcomes and HIV status. Well-trained public health trainees in the University did the data extraction. Cleaning was done to check for the consistency and completeness of the data set. Data were summarized using frequencies and proportions to describe the study population in relation to relevant variables. Prevalence of TB/HIV co-infection was estimated by using the number of TB patients tested positive for HIV as the numerator and the total number of TB patients tested for HIV as the denominator. Bivariate and multivariate logistic regression were used to identify significant predictors of HIV infection. The degree of association between dependent and independent variables was assessed using odds ratios (OR) with 95% confidence intervals (CI), and statistical significance was set at *p*-value <0.05.

### Definitions of terms

**New TB patient** refers to a patient who has never had treatment for tuberculosis or who has taken antituberculosis drugs for less than 1 month.

**Pulmonary Tuberculosis (PTB)** refers to a patient with tuberculosis disease involving the lung parenchyma.

**Smear positive PTB** refers to a patient with at least one sputum smear positive for acid fast bacilli (AFB), or one sputum smear positive for AFB plus radiographic abnormalities consistent with active pulmonary tuberculosis; or one sputum specimen positive for AFB plus culture specimen positive for *Mycobacterium tuberculosis*.

**Smear negative PTB** refers to a patient with two negative sputum smears for AFB but radiological abnormality consistent with active TB or failure to respond to antibiotics treatment or one which health worker or clinician has diagnosed TB and decided to treat the patient with full course of anti TB drugs.

**Extra pulmonary TB (EPTB)** refers to patients with TB in any organ other than the lungs verified by histopathology.

### Ethical statement

This study utilized routine service delivery data for the purposes of programme monitoring and evaluation. The data analysed did not include identifiers of the patients and was exempted from ethical approval. The University of Health and Allied Sciences, School of Public Health’s scientific review committee ruled that this study do not require formal ethical approval.

## Results

There were 1772 TB cases registered during the period under study. Of these, 1167 (65.9%) were males and 605 (34.1%) were females; 752 (42.4%) were between 30 and 49 years of age; 87 (4.9%) were <15 years, while 192 (10.8%) were 70 years and above. The age range of patients was 1–100 years with median age (interquartile range (IQR)) of 43 (24). One thousand and twenty five (58%) were smear positive pulmonary TB cases, 672 (37.9%) were smear negative pulmonary TB cases, and 66 (3.7%) extra-pulmonary TB cases. With respect to TB category, 1637 (92.4%) were new TB cases, while 39 (2.2%), and 30 (1.7%) were relapse and treatment failure cases respectively. Of the total 1772 patients, 448 (25.3%) were registered in Ho Municipal, while 395 (22.3%) and 275 (15.5%) were registered in Hohoe and Kadjebi respectively. Kpando and Keta recorded 327 (18.5%) cases each (Table 1).

**Table 1:** General characteristics of study subjects, 2012–2015

Characteristic	2012 (*N* = 579) n (%)	2013 (*N* = 433) n (%)	2014 (*N* = 340) n (%)	2015 (*N* = 420) n (%)	All years (*N* = 1772) n (%)
*Age group (years)* ^*a*^					
0–14	43 (7.4)	22 (5.1)	13 (3.8)	9 (2.1)	87 (4.9)
15–29	98 (16.9)	69 (15.9)	49 (14.4)	60 (14.3)	276 (15.6)
30–49	240 (41.5)	179 (41.3)	154 (45.3)	179 (42.6)	752 (42.4)
50–69	132 (22.8)	116 (26.8)	93 (27.4)	122 (29.1)	463 (26.1)
≥ 70	66 (11.4)	47 (10.9)	31 (9.1)	48 (11.4)	192 (10.9)
*Sex*					
Male	374 (64.6)	276 (63.7)	221 (65.0)	296 (70.5)	1167 (65.9)
Female	205 (35.4)	157 (36.3)	119 (35.0)	124 (29.5)	605 (34.1)
*Patient category* ^*b*^					
New	547 (94.5)	393 (90.8)	322 (94.7)	375 (89.3)	1637 (92.4)
Transferred in	1 (0.2)	3 (0.7)	1 (0.3)	0	5 (0.3)
Return after default	5 (0.9)	3 (0.7)	1 (0.3)	7 (1.7)	16 (0.9)
Treatment failure	4 (0.7)	9 (2.1)	5 (1.5)	12 (2.9)	30 (1.7)
Relapse	6 (1.0)	11 (2.5)	10 (2.9)	12 (2.9)	39 (2.2)
Other	15 (2.6)	15 (3.2)	1 (0.3)	3 (0.7)	33 (1.9)
*TB classification* ^*c*^					
Pulmonary positive	262 (45.3)	240 (55.4)	224 (65.9)	299 (71.2)	1025 (57.8)
Pulmonary negative	279 (48.2)	183 (42.3)	109 (32.1)	101 (24.1)	672 (37.9)
Extra-pulmonary	37 (6.4)	10 (2.3)	7 (2.1)	12 (2.9)	66 (3.7)
*Treatment District*					
Ho	194 (33.5)	123 (28.4)	67 (19.7)	64 (15.2)	448 (25.3)
Hohoe	127 (21.9)	97 (22.4)	95 (27.9)	76 (18.1)	395 (22.3)
Kadjebi	51 (8.8)	91 (21.0)	43 (12.7)	90 (21.4)	275 (15.5)
Keta	118 (20.4)	47 (10.9)	53 (15.6)	101 (26.0)	327 (18.5)
Kpando	89 (15.4)	75 (17.3)	82 (24.1)	81 (19.3)	327 (18.5)

Missing values for age^a^, patient category^b^, and TB classification^c^; 2 (0.1%); 12 (0.7%); and 9 (0.5%) respectively

### HIV testing and prevalence of HIV among TB patients

**Fig. 2:**
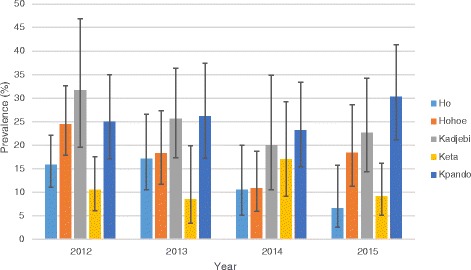
Annual prevalence of TB/HIV co-infection stratified by district

Of the 1772 TB cases registered during the study period, 1633 (92.2%) were tested for HIV. The proportion of TB cases tested for HIV was more than 90% for all districts except Ho and Kadjebi where 85% and 81.8% of cases registered during the period were tested respectively. Of the 1633 cases tested for HIV, 297 (18.2%; 95% CI: 16.4–20.1) were HIV positive. The prevalence was highest in Kpando municipal (26.0%; 95% CI: 21.5–31.2) followed by Kadjebi district (24.9%; 95% CI: 19.7–30.9) and lowest in Keta municipal (10.8%; 95% CI: 7.9–14.7) as shown in Table 2 and Fig. 2.

**Table 2:** HIV testing and prevalence of HIV among TB patients from 2012 to 2015

District	#TB cases registered	HIV test n (%)	HIV Positive n (%)	95%CI
Ho	448	381 (85.0)	52 (13.7)	10.6–17.5
Hohoe	395	389 (98.5)	72 (18.5)	15.0–22.7
Kadjebi	275	225 (81.8)	56 (24.9)	19.7–30.9
Keta	327	323 (98.5)	35 (10.8)	7.9–14.7
Kpando	327	315 (96.3)	82 (26.0)	21.5–31.2
**All Districts**	**1772**	**1633 (92.2)**	**297 (18.2)**	**16.4–20.1**

### Prevalence of TB/HIV co-infection stratified by sex and age

**Fig. 3:**
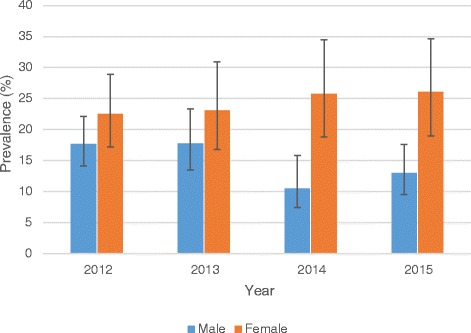
Annual prevalence of TB/HIV co-infection stratified by sex

**Fig. 4:**
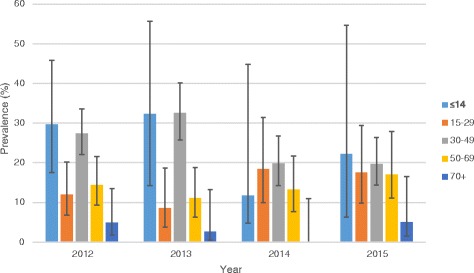
Annual prevalence of TB/HIV stratified by age group

The overall prevalence of TB/HIV co-infection was significantly higher (24.1%; 95% CI: 20.8–27.7) among females compared to males (15.1%; 95% CI: 13.1–17.4) (*p* < 0.001). With regards to age, the prevalence was highest (27.0%; 95% CI: 18.2–38.1) among patients <15 years of age and lowest (3.5%; 95% CI: 1.6–7.4) among patients ≥70 years (*p* < 0.001). Fig. 3 shows the trend of prevalence of TB/HIV co-infection stratified by sex whereas Fig. 4 represents the prevalence of TB/HIV by age groups.

### Trends of TB/HIV co-infection stratified by district

The prevalence of TB/HIV co-infection was stable during the four-year study period. In the year 2012, 105 (19.4%; 95% CI: 16.3–22.9) TB patients were positive for HIV. The prevalence remained fairly stable (19.8%; 95% CI: 16.0–24.2) in the year 2013 and decreased to 15.8% (95% CI: 12.3–20.1) in 2014 and subsequently rose slightly to 17.0% (95% CI: 13.6–21.1; *p* = 0.424) in the year 2015.

### Treatment outcomes of TB only and TB/HIV co-infected patients

Treatment outcomes significantly differed among TB cases and TB/HIV co-infected patients. Nearly 90% (95% CI: 88.1–91.5) of only TB cases evaluated were successfully treated, compared to 77% (95% CI: 71.7–81.7) among TB/HIV co-infected patients (*p* < 0.001). TB/HIV co-infected patients were about 3 times more likely to die before completing treatment compared to TB only patients. Defaulter rate was also highest (9; 3.3%) among co-infected patients compared to 21 (1.7%) among patients with TB only. However, treatment failure rate was about 2 times more among patients with TB only compared to TB/HIV co-infected patients as shown in Table 3.

**Table 3:** Treatment outcomes among patients with TB only compared to TB/HIV co-infected patients

Type of patient	Successful	Unsuccessful			Total evaluated N (%)
	Cured +completed n (%)	Died n (%)	Defaulted n (%)	Treatment Failure n (%)	
TB only	1096 (89.9)	77 (6.3)	21 (1.7)	25 (2.1)	1219 (77.9)
TB/HIV	208 (77.0)	50 (18.5)	9 (3.3)	3 (1.1)	270 (22.1)
Total	1304 (87.6)	127 (8.5)	30 (2.0)	28 (1.2)	1489 (100)

*p* < 0.001

### Predictors of HIV infection among TB patients

In this study, it was observed that female TB patients were significantly more likely to be HIV positive compared to males (OR: 1.79; 95% CI: 1.38–2.31; *P* < 0.001) on bivariate analysis. Also patients’ age, TB classification, and treatment district were significantly associated with positive HIV result.

In the adjusted analysis, patients who were ≥70 years old had 91% lower odds of being infected with HIV (AOR: 0.09; 95% CI: 0.04–0.26; *P* < 0.001) than patients who were less than 15 years old. Additionally, female TB patients were about 2 times more likely to be HIV positive compared to male patients (AOR: 1.89; 95% CI: 1.44–2.49; *P* < 0.001). With regards to TB classification, smear negative pulmonary TB patients were 84% more likely to be HIV positive (AOR: 1.84; 95% CI: 1.37–2.47; *P* < 0.001) than smear positive pulmonary TB patients. However, the odds of HIV infection was not significantly different among extra-pulmonary TB patients compared to the reference group. TB Patients registered in Keta municipality had 62% lower odds of being HIV positive (AOR: 0.62; 95% CI: 0.38–0.99; *P* = 0.047), compared to those registered in Ho municipality. Conversely, patients registered in Hohoe, Kadjebi, and Kpando were more likely to be HIV positive (AOR: 1.69; 95% CI: 1.13–2.54; *P* = 0.01), (AOR: 2.29; 95%CI: 1.46–3.57; *P* < 0.001), (AOR: 2.15; 95% CI: 1.44–3.21; *P* < 0.001) respectively compared to the reference district (Table 4).

**Table 4:** Predictors of HIV among TB patients in Volta Region, Ghana from 2012 to 2015

Characteristic	TB/HIV n (%)	OR (95% CI)	*p*-value	AOR (95% CI)	*p*-value
*Age group (years)*			0.001		
0–14	20 (27.0)	1		1	
15–29	35 (13.7)	0.43 (0.23–0.79)		0.52 (0.27–1.01)	0.054
30–49	176 (25.0)	0.90 (0.53–1.55)		1.15 (0.65–2.05)	0.632
50–69	60 (14.1)	0.44 (0.25–0.79)		0.52 (0.28–0.96)	0.036
≥ 70	6 (3.5)	0.09 (0.04–0.26)		0.09 (0.04–0.26)	0.001
*Sex*			0.001		
Male	162 (15.1)	1		1	
Female	135 (24.1)	1.79 (1.38–2.31)		1.89 (1.44–2.49)	0.001
*Patient category*			0.931		
New	275 (93.2)	1			
Transferred in	0	-			
Defaulter	3 (1.0)	1.50 (0.40–5.58)			
Treatment failure	6 (2.0)	1.13 (0.46–2.78)			
Relapse	7 (2.4)	1.09 (0.47–2.51)			
Other	4 (1.8)	0.72 (0.25–2.09)			
*TB classification*			0.0015		
Pulmonary positive	151 (15.8)	1		1	
Pulmonary negative	139 (22.5)	1.54 (1.19–1.99)		1.84 (1.37–2.47)	0.001
Extra-pulmonary	5 (10.4)	0.62 (0.24–1.59)		0.58 (0.22–1.55)	0.279
*Treatment District*			0.001		
Ho	52 (13.7)	1		1	
Hohoe	72 (18.5)	1.44 (0.97–2.12)		1.69 (1.13–2.54)	0.011
Kadjebi	56 (24.9)	2.09 (1.38–3.19)		2.29 (1.46–3.57)	0.001
Keta	35 (10.8)	0.77 (0.49–1.21)		0.62 (0.38–0.99)	0.047
Kpando	82 (26.0)	2.23 (1.51–3.28)		2.15 (1.44–3.21)	0.001
*Year registrated*			0.419		
2012	105 (19.4)	1			
2013	73 (19.8)	1.02 (0.73–1.43)			
2014	53 (15.8)	0.78 (0.54–1.12)			
2015	66 (17.0)	0.85 (0.61–1.19)			

*OR* odds ratio, *AOR* adjusted odds ratio

## Discussion

### HIV testing for TB patients and TB/HIV co-infection rates

Systematic screening for HIV among TB patients is recommended by WHO as an essential component of the TB care package. This study established that more than 9 in 10 TB patients notified during the period had documented HIV test result, higher than the WHO’s global estimate of 55% in 2015 and that of the African Regions (81%) [1]. This shows that HIV screening practices among TB patients is good in this part of the world and should be sustained. Similar finding was reported in a study in Ethiopia [10].

Regional and countries prevalence studies on TB/HIV co-infection showed varied values ranging between 2.9 and 72.3%, with pooled prevalence of 23.5% [11]. The overall HIV seroprevalence in TB patients in this study is higher than the current global estimate of 15% but much lower than the estimate from the African Region (36%) [1] and the national average of 24% [12] but is comparable to findings from studies done in India (18.9%) and Brazil (19%) [13, 14].

Overall, the global prevalence of TB/HIV co-infection has been falling since 2008 [1]. Conversely, this study identified a stable TB/HIV co-infection in the study districts during the years 2012 to 2015. This suggests that additional efforts to strengthen TB/HIV collaborative activities are required in order to reduce the burden of HIV in TB patients. However, given that we used only 4 years in our analysis, we recommend further studies which incorporate data of several years before a firm conclusion can be drawn.

### Predictors of HIV infection in TB patients

In this study, TB/HIV coinfection rate was significantly higher in female patients than in males. This finding is consistent with a number of studies that showed that females bear a disproportionate burden of HIV infections in sub-Saharan Africa [15–18]. The factors contributing to this could include lower socioeconomic position of women [18] as well as biology, sexual behaviour and socially constructed gender differences between men and women in roles and responsibilities, access to resources and decision-making power [18, 19]. Our finding is however contrary to findings from a study in Northwest Ethiopia [10] but similar to report from Northeastern Ethiopia [20].

A high rate of TB/HIV co-infection was also observed in patients <15 years of age. This is in contrast to several studies that have shown TB/HIV coinfection rates to be higher in persons aged 25–45 years [10, 21, 22] representing the sexually active age group. The high prevalence in persons <15 years could imply early indulgence in sexual activities by this age group in the study districts but this needs to be thoroughly investigated.

Among patients with pulmonary TB, those with sputum smear negative were about 2 times more likely to be co-infected with HIV. This finding concords with that of a previous report, which showed that, in patients with HIV/AIDS, especially in the late stage of HIV infection, TB is often atypical in presentation and has low sputum smear positivity in pulmonary TB, which could result in delayed diagnosis of TB [23].

### Treatment outcomes of HIV-positive and HIV-negative TB patients

TB/HIV co-infected patients in this study had more unsuccessful treatment outcomes than patients with TB only and were more likely to die before completing treatment. Unsuccessful treatment outcomes associated with TB/HIV coinfection have been shown by several studies [21–24]. The reason for the poor outcomes in this group could be due to drug interactions between the rifamycins (rifampin or rifabutin) and some antiretroviral agents [25] as well as malabsorption of anti-TB drugs among patients with advanced HIV [26], leading to low serum concentrations of drugs and therefore to unsuccessful treatment outcomes [27]. In addition, the high death rate among TB/HIV co-infected patients could be due to alternation of the clinical manifestation of TB and lack of a rapid and sensitive TB diagnostic test for diagnosing TB in HIV patients leading to delayed diagnosis and treatment [28–30] that could eventually lead to death. The clinical alteration of TB in HIV-positive patients could also explain they were more likely to be smear negative as seen in this study. In contrast, treatment failure rate was twice more higher in patients with only TB compared to TB/HIV co-infected patients. Several reasons could account for this; it could be that some TB patients resort to traditional/herbal treatment, poor adherence to treatment and some could even have drug resistant TB [31, 32] and therefore will not respond to first line anti-TB drugs but this needs to be evaluated further in another study.

This study was retrospective in design and based only on data that was available in public health records. We could not independently corroborate the accuracy of these records, nor could we collect additional data needed to confirm or refute our findings. In particular we could not differentiate between TB/HIV co-infected patients on ART and those not on ART, and therefore, we could not assess its effect on our findings. Though a limitation, it calls for linkage of databases between TB and HIV control programs to ensure completeness of data for TB/HIV co-infected patients at all levels of the health care system. This will improve analysis of data that can provide accurate information for effective planning of targeted interventions. Despite this limitation, the study did provide useful information on the burden of HIV among TB patients in five districts of the Volta region. Findings may be useful to TB program managers and policy makers in implementing interventions to improve control of these infections.

## Conclusion

The proportion of TB/HIV co-infection in the study districts was quite high and associated with females, persons aged <15 years. Additionally, HIV-negative TB patients had better treatment outcomes compared to HIV-positive patients. There is the need for targeted interventions like awareness creation for females and persons aged <15 years. Factors accounting for unsuccessful treatment outcomes in co-infected patients should be identified and addressed to improve treatment outcomes and prevent deaths in this vulnerable population. Also, factors responsible for treatment failure in HIV-negative TB patients in this setting should be investigated.

## Abbreviation

AFB: Acid fast bacilli
AIDS: Acquired immune deficiency syndrome
ART: Antiretroviral therapy
CI: Confidence interval
DOTS: Directly observed treatment short-course
HIV: Human immune-deficiency virus
NTP: National TB control programme
TB: Tuberculosis
WHO: World Health Organization
